# Effects of a Rehabilitation Program Using a Patient-Personalized Exergame on Fear of Falling and Risk of Falls in Vulnerable Older Adults: Protocol for a Randomized Controlled Group Study

**DOI:** 10.2196/24665

**Published:** 2021-08-26

**Authors:** Nolwenn Lapierre, Nathavy Um Din, Manuella Igout, Joël Chevrier, Joël Belmin

**Affiliations:** 1 Hôpital Charles Foix Assistance Publique-Hôpitaux de Paris AP-HP.Sorbonne Université Ivry-Sur-Seine France; 2 Centre de Recherches Interdisciplinaires Université Paris Descartes Paris France; 3 Faculté de Médecine Sorbonne Sorbonne Université Paris France

**Keywords:** older adult, fall, fear of falling, exergame, randomized controlled trial, psychomotor therapy, rehabilitation, fear, risk, elderly, protocol, therapy

## Abstract

**Background:**

Older adults often experience physical, sensory, and cognitive decline. Therefore, they have a high risk of falls, which leads to severe health and psychological consequences and can induce fear of falling. Rehabilitation programs using exergames to prevent falls are being increasingly studied. Medimoov is a movement-based patient-personalized exergame for rehabilitation in older adults. A preliminary study showed that its use may influence functional ability and motivation. Most existing studies that evaluate the use of exergames do not involve an appropriate control group and do not focus on patient-personalized exergames.

**Objective:**

This study aims to evaluate the effects of Medimoov on risk of falls and fear of falling in older adults compared with standard psychomotor rehabilitation.

**Methods:**

This is a serial, comparative, randomized controlled group study. Both groups (n=25 in each) will receive psychomotor rehabilitation care. However, the methods of delivery will be different; one group will be exposed to the Medimoov exergame platform, and the other only to traditional means of psychomotor rehabilitation. The selection criteria will be (1) age of 65 years or older, (2) ability to answer a questionnaire, (3) ability to stand in a bipedal position for at least 1 minute, (4) score of 13 or greater on the Short Fall Efficacy Scale, and (5) stable medical condition. An evaluation will be made prior to starting the intervention, after 4 weeks of intervention, and at the end of the intervention (after 8 weeks), and it will focus on (1) risk of falls, (2) fear of falling, and (3) cognitive evaluations. Physical activity outside the session will also be assessed by actimetry. The outcome assessment will be performed according to intention-to-treat analysis.

**Results:**

The protocol (2019-11-22) has been approved by the Comité de Protection des Personnes Nord-Ouest I–Université de Rouen (2019-A00395-52), which is part of the French national ethical committee. The study received funding in February 2020. As of October 2020 (submission date), and due to the context of the COVID-19 pandemic, a total of 10 participants out of 50 had been enrolled in the study. The projected date for the end of the data collection is December 2021. Data analyses have not been started yet, and publication of the results is expected for Spring 2022.

**Conclusions:**

The effects of psychomotor rehabilitation using the Medimoov exergame platform on the risk and fear of falls will be evaluated. This pilot study will be the basis for larger trials.

**Trial Registration:**

ClinicalTrials.gov NCT04134988; https://clinicaltrials.gov/ct2/show/NCT04134988

**International Registered Report Identifier (IRRID):**

DERR1-10.2196/24665

## Introduction

The ageing population has a higher risk of developing sensory, physical, mental, and cognitive disorders, which increase their risk of falls [1,2]. Physical disability is highly prevalent in older adults and encompasses mobility and balance impairment. Cognitive alterations also induce walking difficulties, leading to increased risk of falls [2,3]. Each year, 20% to 30% of older adults fall [1]. Of these, 25% to 50% will relapse within the year, which aggravates the problem by the multiplication of injuries [1]. Falls lead to numerous and serious health consequences, loss of independence, and institutionalization [1,4]. In addition, falls and balance impairment may induce fear of falling, which may lead to social isolation, anxiety, and depression as well as decreased mobility, physical disability, and falls [5,6]. Fear of falling encompasses fall-related fear, fall-related self-efficacy, and balance confidence [7]. In community-dwelling older adults (≥75 years old), 41.5% experience fear of falling. In long-term care, this prevalence is higher: 63% of institutionalized older adults express a fear of falling. A recent literature review shows that community-dwelling older adults (≥65 years old) score an average of 15 on the Short Fall Efficacy Scale–International (Short FES-I), which measures the fear of falling; this score indicates a moderate fear of falling [8].

Fall prevention programs can reduce the risk of falls if they combine different exercise categories [9,10]. The literature review of Vieira et al (2016) [3] and the quasi-experimental study of Pereira et al (2014) [10] with 506 community-dwelling older adults demonstrate that benefiting from regular physical activities is associated with an improvement in physical abilities and balance. The systematic review and meta-analysis of Tricco et al (2017) [9], which included 54 studies and 41,596 participants, confirmed these results and added that exercise combined with vision assessment and treatment as well as environmental modification are effective in fall prevention [9]. In addition, fall prevention programs should target cognitive function improvement [2]. Cerebral plasticity and cognitive functions are essential for motion and balance [2]. Among executive functions, inhibition is particularly relevant to fall prevention because poorer results of response inhibition tests are associated with future risk of falls [11]. Cognitive inhibition suppresses irrelevant information from the working memory and facilitates automatic response, which may explain its importance for preventing the risk of falls, especially during mobility tasks involving distraction (eg, walking outside) [11]. Adapting the rehabilitation to the patient’s cognitive status is paramount to prevent falls [12] and to allow older adults to focus on their rehabilitation programs. Psychomotor therapists are among the professionals recommended by the Haute Autorité de Santé, the French national health agency, to safely administer physical exercise programs to older adults [13]. These therapists possess skills and expertise in intervening in the psychomotor dimensions of falls, namely, the motor, sensory, and cognitive aspects [14].

In rehabilitation, exergames are increasingly being studied, especially exergames using Kinect sensors (Microsoft Corporation) [15-18]. According to Tanaka et al [18], “exergaming platforms are designed to track body motion or body reactions and provide both fun and exercise for game players.” Several exergames have been designed for the rehabilitation of older adults, and some of them were found to be effective in studies with before-and-after designs or randomized controlled trials [19,20]. Their safety was deemed good: no adverse effects related to their use were identified [21]. We did not find any studies that compared the effects of patient-personalized exergame-assisted rehabilitation with those of traditional rehabilitation programs [21]. Studies should explore not only the efficacy of an exergame-assisted rehabilitation program, but also its acceptability [21]. Adherence to and engagement in a rehabilitation program are at the heart of its effectiveness and therefore should be considered when evaluating the exercise rehabilitation program [21]. There is no consensus in the literature regarding the adherence of older individuals to rehabilitation programs with exergames compared to programs without exergames [21]. However, some experimental studies with older adults have shown better adherence in a rehabilitation group with exergames than in the control group [22,23]. Exergames are considered to be enjoyable and have the potential to increase motivation and reduce fear of falling. Therefore, they are of particular interest in engaging older people who are afraid of falls in exercise programs.

Medimoov (NaturalPad) [24] is a movement-based video game for rehabilitation of older adults that allows the therapist to choose the movements the patient will perform to interact with the game. The difficulty and the speed of the game can be defined by the therapist, allowing for a fine-tuned personalization of the sessions according to the patient’s progress. A preliminary study showed that using Medimoov’s personalized activities may positively influence older adults’ functional ability and motivation [15]. Most existing studies evaluating the use of exergames do not involve an appropriate control group (ie, a control group following the standard rehabilitation program) [17,21] or do not involve an exergame specifically designed for rehabilitation of older adults that allows for the personalization of the sessions [21,22,25]. Thus, this study aims to evaluate the effects of Medimoov on older adults with fear of falling compared with standard psychomotor rehabilitation as prescribed by physicians. Two aspects of the rehabilitation will be explored: the preventive function (decreased risk of falls) and the therapeutic function (decrease of fear of falling and increase of motivation during rehabilitation).

The main objective is to evaluate the efficacy of exergames played on the Medimoov platform on fear of falling and risk of falls. The secondary objective is to assess the effect of the exergame intervention on the participants’ executive functions during the study. Another secondary objective is to explore the perception of exergame-assisted rehabilitation by the participants of the intervention group. The hypothesis is that exergames in rehabilitation can lead to decreased fear of falling and risk of falls due to an improvement of balance, physical functions, and executive functions, including an improvement in cognitive inhibition.

## Methods

### Design

This study is an open, randomized controlled study in parallel groups with blind data collection for the evaluation criteria. The protocol follows the SPIRIT (Standard Protocol Items: Recommendations for Interventional Trials) guidelines [26,27].

### Participants and Recruitment

The research project will be conducted in two intermediate-care wards for older adults at the Charles Foix Hospital, a French geriatric hospital. Guidelines to calculate the sample size do not exist in the literature yet; thus, the sample size has been estimated based on previous studies evaluating fear of falling–focused interventions [28,29]. A sample size of 20 participants per group is often reported. A total of 25 participants per group will be recruited to factor in a potential 20% withdrawal or other variations. Recruitment will be conducted among hospitalized patients in intermediate-care rehabilitation hospital wards. The inclusion criteria will be as follows: (1) age of 65 years or older, (2) ability to understand and answer short and simple questions (as determined by the clinical interview), (3) ability to stand upright in a bipedal position, feet together, for longer than 1 minute with or without a technical aid, (4) a score of 13 or greater on the FES-I [30] (moderate or severe fear of falling), (5) stable medical condition, and (6) ability to give written consent. The exclusion criteria will be as follows: (1) cannot correctly distinguish the elements on the screen, (2) in palliative care, (3) under a legal protection measure, (4) obtained a score <16 on the Mini Mental State Examination (MMSE) [31], and (5) recently underwent orthopedic surgery (<2 months); the latter is an exclusion criterion because it requires specific rehabilitation programs [32]. The MMSE will be used for screening purposes only to exclude patients with severe cognitive disorders that would prevent them from participating. Participation may be interrupted at the participant’s request or if the participant experiences an adverse health event (eg, stroke). All patients hospitalized in the rehabilitation ward at the beginning of the study will be screened for eligibility by their physicians, and if eligible, will be asked to participate after a detailed presentation of the protocol by a postdoctoral fellow or a research professional. The eligibility of the patients admitted to hospital after the start of the study will be verified. Participant recruitment will be ongoing until the sample number (N=50) is reached. Eligible patients will receive a letter of information. A random selection will be conducted if the number of eligible patients is higher than the number of required participants.

### Randomization

After the randomization, patients will be allocated to an intervention group (rehabilitation program with the Medimoov platform) or to a control group (traditional psychomotor therapy sessions). The randomization will be conducted directly after the signature of the consent form. To ensure concealed allocation [33], each participant will be assigned a code used for the randomization. As the participants will not all be identified before the randomization, block randomization will be conducted (rather than stratification) to control the sample size of each group using the “blockrand” function of RStudio software (RStudio PBC).

### Ethical Considerations

The protocol (2019-11-22) has been approved by the Comité de Protection des Personnes Nord-Ouest I–Université de Rouen (2019-A00395-52), which is part of the French national ethical committee. Written informed consent will be collected from each older adult before their participation. The study is registered on ClinicalTrials.gov under the number NCT04134988 (2019/10/22) and in the Commission Nationale de l'Informatique et des Libertés, which verifies the legality and security of computerized personal data in France.

### Rehabilitation Programs

Both rehabilitation programs (intervention and control) will be consistent with the prescription of the participants’ physicians. For each group, the program will be guided by a psychomotor therapist and will last 8 weeks, with 35-minute individual sessions twice per week. According to professional practices in psychomotor therapy, the psychomotor therapist will ensure the progress of each group’s sessions by adapting their proposals to the participant.

#### Intervention Group: Medimoov Sessions

**Figure 1:**
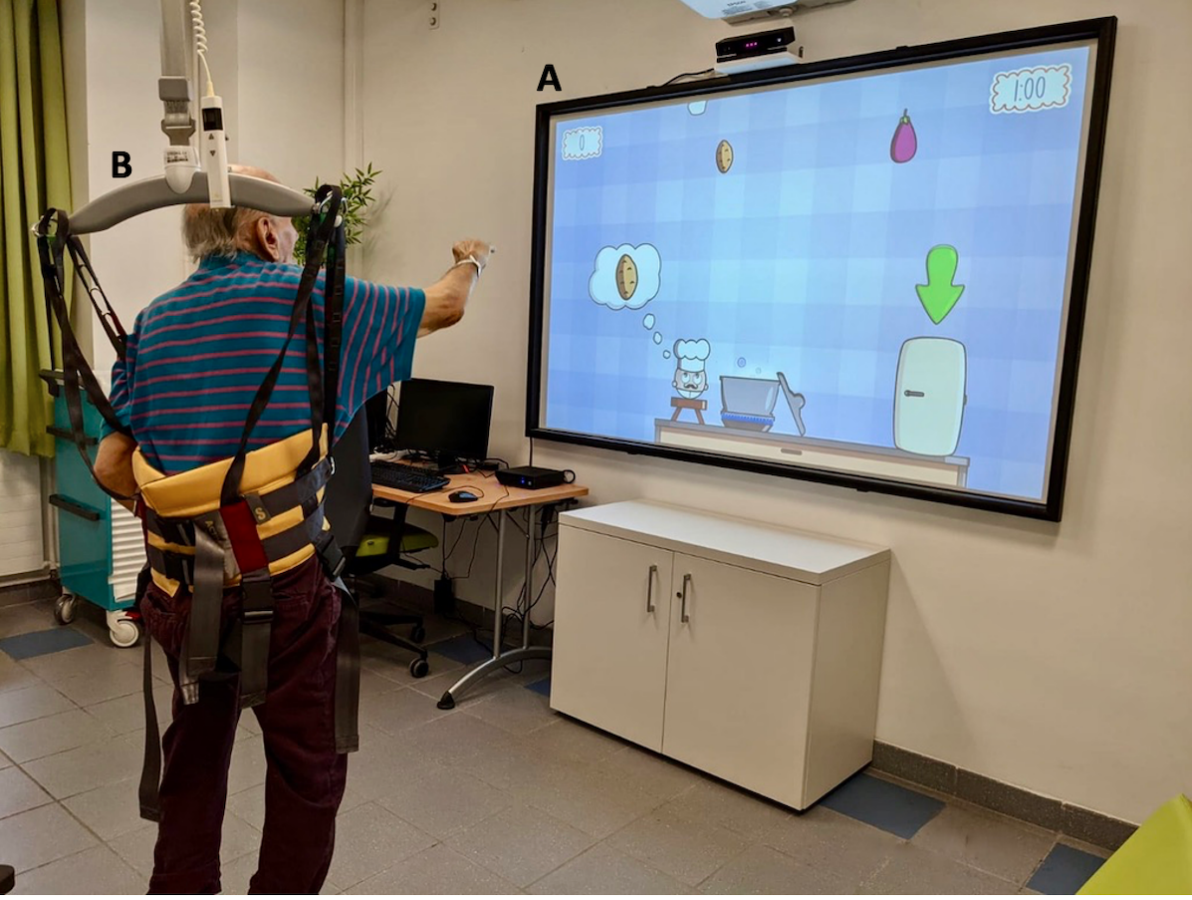
(A) The large screen displays an exergame. The Kinect camera is positioned above the screen to capture the movements of the participant; the computer with the Medimoov platform is on the left of the screen. (B) The harness protects the participants from injury if they fall during the session.

**Figure 2:**
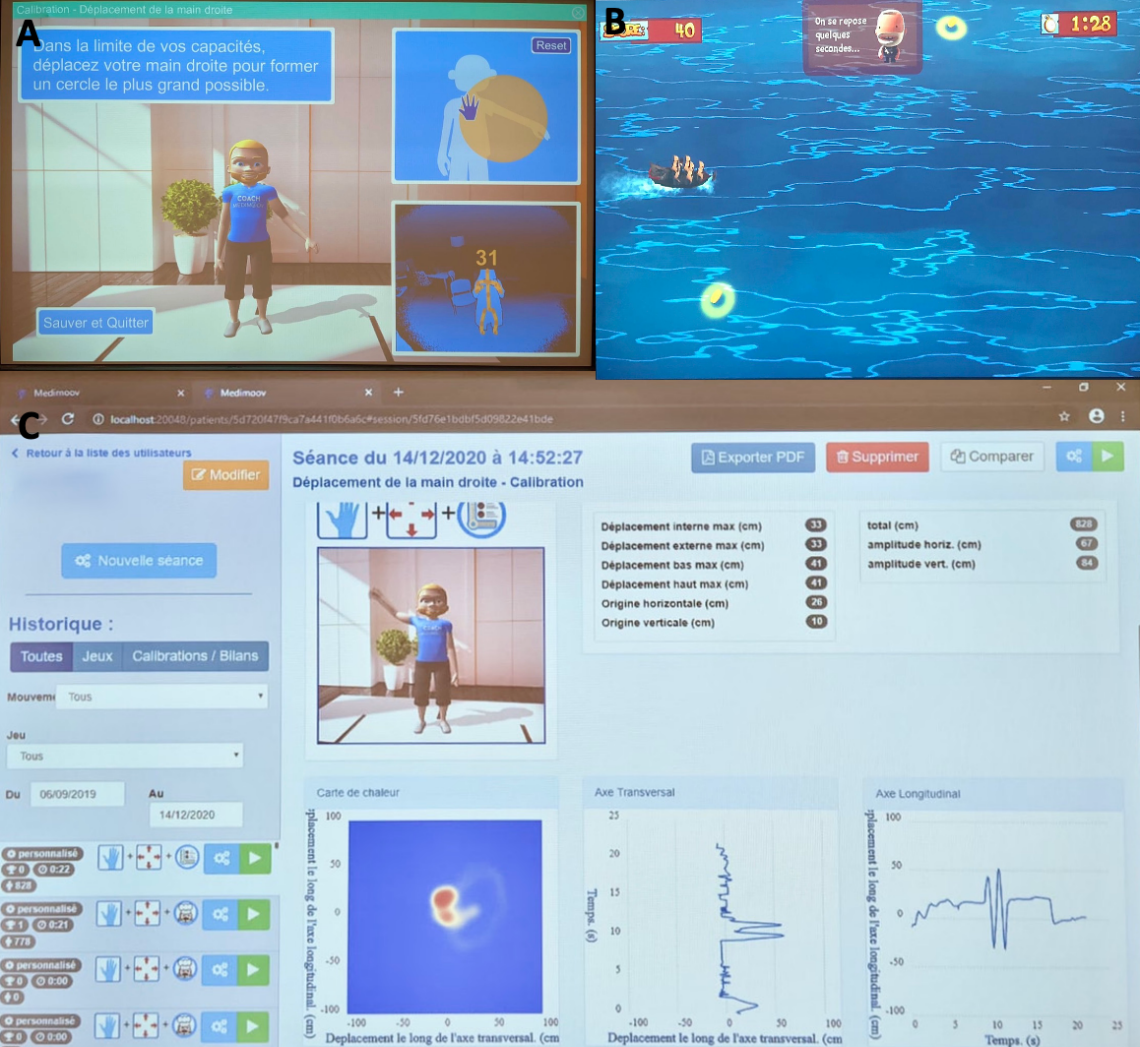
(A) Medimoov allows calibration of its games according to the participant’s abilities. The instructions for the movements to be performed are displayed on the screen. The orange circle indicates the participant’s maximum range of motion, and a feedback image reconstructing the movements captured by the Kinect is presented at the bottom of the screen. (B) Several games can be played. For one of the games, the participant leads a pirate ship to recover gold coins while avoiding rocks and enemy fire. (C) Each participant has a personal profile in which the psychomotor therapist can access the participant’s session and performance history.

The Medimoov gaming platform [15] is a physio-gaming medical device that works using functional and postural rehabilitation software. The therapy requires a computer equipped with Medimoov, a Kinect camera, a projection screen, and an immersive sound system (Figure 1). Each participant has their own profile in which their age, gender, and physical capacities (eg, their amplitude of movement) are stored (Figure 2). Each game’s required movements are adjusted to the participant’s capacities with personalized calibration. Medimoov offers 14 exercises (eg, upper limb work, sit-to-stand exercises) within 6 different games that can be configured according to specific conditions (eg, spatial neglect) and different cognitive statuses (eg, simplified decor to avoid distraction for people with limited attention capacities). The length of each game can also be configured according to the participant’s stamina.

The program will last 8 weeks, with biweekly individual sessions of 35 minutes for a total of 16 sessions. The personal calibration and the creation of the participant’s profile will be performed during the first session. The psychomotor therapist will systematically offer the use of a harness before the beginning of the session. This harness, which is hung from a rail system, secures the person while they are standing and during motor activity. The psychomotor therapist will adapt their proposals to the participant according to his/her professional practice. The sessions will be conducted in the following order: (1) sessions 1 to 4, work on the cervical spine and upper limbs while seated; (2) sessions 5 to 8, in a sitting position, work on limb movement amplitude and precision and movement of the spine as a whole; (3) sessions 9 to 12, work on the spine as a whole with a special focus on the cervical spine with static and dynamic balance in the standing position; and (4) sessions 13 to 16, work on the movement amplitude and precision with static and dynamic balance in the standing position.

#### Control Group: Traditional Psychomotor Therapy Sessions

Parallel to the intervention group, the program will last 8 weeks, with biweekly sessions of 35 minutes for a total of 16 sessions. This traditional psychomotor therapy rehabilitation program will be the one that is usually conducted in the ward. As in the intervention group, the psychomotor therapist will ensure the progress of the sessions by adapting their proposals to the participant according to their professional practice. The individual sessions will be based on the following progression. (1) Sessions 1 to 4, body waking exercises, soft gymnastics with cervical spine and upper limb mobilizations in a seated position; (2) sessions 5 to 8, upper and lower limb movement coordination exercises in a seated position and movement of the spine as a whole; (3) sessions 9 to 12, static and dynamic balance exercises with work on the spine as a whole with a special focus on the cervical spine in a standing position; (4) sessions 13 to 16, in the standing position, psychomotor trails (movement amplitude and precision).

### Study Outcome and Measures

Sociodemographic data will be collected from the participants’ medical records and will include the following: age, gender, highest education level, living conditions (eg, alone or with relatives, at home or in long-term care), reason for hospitalization, the presence of visual or hearing impairment, and the number of falls during the past 12 months. Clinical evaluations will focus on the following: (1) balance and risk of falls (Score Physical Performance Battery [SPPB] test, One-Leg Stand test, Timed Up and Go [TUAG]), (2) fear of falling (Short FES-I) and (3) cognitive evaluations (Stroop test, Trail Making Test Parts A and B). Wrist actimetry will be used to assess all older adults’ spontaneous motor activity outside rehabilitation sessions (eg, engaging in their activities of daily living, walking indoors in their room and the common areas of the ward, or walking outdoors in the hospital garden). The study procedure and assessment schedule are presented in Table 1.

**Table 1:** The study procedure is presented following the SPIRIT (Standard Protocol Items: Recommendations for Interventional Trials) template.

Phase and time point (week)			Enrollment		Allocation		Postallocation					End	
				Week 0		Week 1			Week 4		Week 8		
**Enrollment**													
	Eligibility screening	✓											
	Informed consent	✓											
	Allocation			✓									
**Interventions**													
	Medimoov or standard psychomotor therapy												
**Assessments**													
	Timed Up and Go					✓			✓		✓		
	One-Leg Stand					✓			✓		✓		
	Short Fall Efficacy Scale					✓			✓		✓		
	Stroop					✓			✓		✓		
	Trail Making Test Parts A and B					✓			✓		✓		
	Score Physical Performance Battery					✓			✓		✓		
	Actimetry					✓			✓		✓		
	Satisfaction questionnaire^a^										✓		

^a^This assessment is specific to the intervention group.

### Assessments

#### The FES-I

This 5-minute questionnaire [34] is composed of 7 questions on daily activities and focuses on fear of falling. Each question can be scored between 1 (“Not at all concerned”) to 4 points (“Very concerned”). Compared to the Activities-Specific Balance Confidence Scale and the Geriatric Fear of Falling Measurement, the FES-I had the best internal consistency [35]. The 4-week test-retest fidelity, measured by an intraclass correlation coefficient (*r*), is 0.83 [30]. Although the FES-I lacks responsiveness among nonfrail older adults, it has been chosen here because hospital inpatients are often frail [35]. As this scale is often used in other experimental studies, using it will allow the results of this study to be compared with those of previous studies [36-39].

#### The TUAG test

This test [40] consists of standing up from a chair with armrests, walking 3 meters, turning around, and coming back to sit. The test lasts less than 5 minutes. A shorter time of execution of the test indicates a better balance and stability and a lower risk of falls; the time of execution is thus analyzed as a quantitative value indicating the risk of falls. Risk of falls is identified if the duration of the test is longer than 20 seconds. The 2-week test-retest fidelity, measured by an intraclass correlation coefficient (*r*), ranges between 0.93 and 0.99 [40]. Compared to other assessment tools for risk of falls, such as the One-Leg Stand test, the Functional Reach test, and the Tinetti test, the TUAG has demonstrated better discriminant validity for fall prediction [40]. When compared to the Berg Balance Scale and the Tinetti test, the TUAG test has demonstrated the best sensitivity to change (93%) [41]. It has also demonstrated a higher participation rate than the One-Leg Stand test and the Functional Reach test [40]. For these reasons, it has been chosen as the primary outcome for this study.

#### The SPPB Test

This 5-minute test [42] focuses on static balance, walking, lower-limb strength and risk of falls. This functional assessment has a predictive value for the evolution of the patient's health and autonomy in daily activities after hospital discharge [43]. It provides information on the patient’s balance, quality of walking, and risk of falling. The score varies between 0 and 12; a score <6 indicates low performance and a high risk of falling. The 5-day test-retest fidelity is *r*=0.89 [44]. The test has been chosen for its good responsiveness to change [45].

#### The One-Leg Stand Test

This test [40] consists of standing on one leg for as long as possible on each side. A longer time on one leg indicates a better balance ability. As with the TUAG, the time can be analyzed as a quantitative indicator of risk of falling and quality of walking. For healthy older adults aged between 70 and 79 years, the mean duration is 15.0 seconds (SD 13.9); for healthy older adults aged between 80 and 99 years, the mean duration is 6.2 seconds (SD 9.3). The 2-week test-retest fidelity, measured by an intraclass correlation coefficient (*r*), ranges between 0.93 and 0.99 [40]. Measured by effect size, the One-Leg Stand test has demonstrated a lower sensitivity to change than the TUAG test; thus, it will not be a primary endpoint for this study [40].

#### Actimetry

The participants’ motor activity will be measured continuously for 3 consecutive days by actimetry using a watch that contains accelerometers (Vivago Ltd) worn on the nondominant wrist. Acceleration of movements will be recorded and stored in the watch as arbitrary units per second. For analysis purposes, activity units will be grouped in 3-hour periods during the day (midnight to 2:59 AM, 3 to 5:59 AM, 6 to 8:59 AM, 9 to 11:59 AM, noon to 2:59 PM, 3 to 5:59 PM, 6 to 8:59 PM, and 9 to 11:59 PM). For each participant, the average activity during the 3 days will be calculated based on these 3-hour periods. This innovative exploratory method will not be considered as an endpoint of the study. The aim is to explore how rehabilitation can influence spontaneous mobility in each group and to explore whether it is associated with a decrease in the fear of falling.

#### The Stroop Test

This test [46] is a 5-minute test to evaluate response inhibition capacities. It will be scored by calculating interference indicators and the *z* score, which adjusts the time required to complete the test to the expected performance, taking into account the age and education level of the participant [47]. The *z* score indicates a deficit in response inhibition (score inferior to –1.65), a limitation in response inhibition (score between –1.64 and –0.9), medium inhibition capacities (score between –0.9 and 0.9), superior response inhibition capacities (between 0.9 and 1.64), and highly superior response inhibition capacity (>1.65) [47]. The test-retest fidelity, measured by the intraclass correlation coefficient (*r*), ranges between 0.84 and 0.91 [48], and the meta-analyses of Demakis et al (2004) [49] demonstrated that the Stroop test is sensitive to change.

#### The Trail Making Test Parts A and B

This evaluation [50] focuses on executive functions, including number and letter recognition, cognitive flexibility, visual scan, and motor functions. This evaluation lasts 10 minutes. The score represents the amount of time required to complete parts A and B of the test [50]. Scores are then compared to standards that take into account age and educational level [50]. The test-retest fidelity, measured by the intraclass correlation coefficient (*r*), ranges between 0.76 and 0.94 [51]. In their literature review, Pointrenaud et al (1997) [52] demonstrated that the Trail Making Test is sensitive to change.

#### Documentation of Falls

Adverse events and falls during hospitalization will be documented with their date and severity.

#### Satisfaction Questionnaire

Within the intervention group, participants will answer a satisfaction questionnaire focusing on Medimoov and the exergaming aspect of their rehabilitation. Their answers will be useful to better interpret the results of this pilot study. The satisfaction questionnaire is composed of 10 5-level Likert-type questions; its aim is to evaluate the participants’ experience and their self-confidence after the exergame-based rehabilitation program. The questionnaire takes approximately 2 minutes to complete.

### Study Procedures

Three assessments will be performed by a psychomotor therapist (who is not one of the therapists involved in the rehabilitation program) focusing on physical and cognitive capacities. The first assessment will be performed during the first week, before the start of the rehabilitation program; the second will occur during week 4; and the third will occur during week 8, at the end of the rehabilitation program. Assessments will be blinded; the assessors will not know the assigned group of each participant. To preserve neutrality, assessments will be performed in a different room than the sessions.

#### Endpoints

The primary clinical endpoint is the comparison of the changes in FES-I scores between the two groups. The main secondary endpoint is the comparison of changes in TUAG scores between the groups. Other secondary endpoints are a significantly greater improvement in the One-Leg Stand test, the SPPB test, the Trail Making Test Parts A and B, and the Stroop test in the intervention group and a high level of satisfaction in the intervention group.

#### Statistical Analysis

Sociodemographic data will be descriptively analyzed (mean, standard deviation, percentage, range). Normality will be tested for all quantitative variables. The characteristics of participants of both groups will be compared via a *t* test or a Mann-Whitney test for nonnormally distributed variables. An intention-to-treat analysis will be adopted; data from all included patients will be analyzed even if they withdraw. Independent sample *t* tests for group differences (or Mann-Whitney U tests for nonparametric data) will be used to evaluated differences in endpoints between pre- and postintervention data. Analysis will be carried out using multiple imputation if some participants have withdrawn from the study before the eighth week. Analyses will be performed using RStudio software.

## Results

The study received funding in February 2020. As of October 2020 (submission date), and due to the context of the COVID-19 pandemic, a total of 10 participants out of 50 have been enrolled in the study. The projected date for the end of the data collection is December 2021. Data analyses have not started yet, and publication of the results is expected for Spring 2022.

## Discussion

### Principal Considerations

This study will allow us to evaluate the effect of psychomotor rehabilitation using the Medimoov interactive gaming platform on fear of falling and risk of falls. Most exergames do not allow for the personalization of the games according to the patient’s abilities, which is essential in rehabilitation [53]. An original aspect of Medimoov is that it provides multiple options to personalize each game and follows the progression of the participants; evaluating the effectiveness of this platform will thus provide new rehabilitation perspectives. Moreover, the study by Lister et al (2014) [16] shows that gamification in health often does not follow professional guidelines and recommendations, which leads to mixed results on fear of falling and risks of falls across studies [17,21]. The design of the present study takes into consideration the recommendation of Lister et al (2014), and we will rigorously compare two rehabilitation programs (the program with the Medimoov platform and the traditional psychomotor therapy sessions) that follow the French national guidelines on fall prevention [13]. Thus, this study will address gaps in the literature and will provide knowledge on patient-personalized exergame effectiveness, which has strong potential for practice and policy changes.

Exergames are often considered to facilitate older adults’ motivation to participate in rehabilitation programs [19-21,54]. This study will allow us to explore this assumption and compare participant adherence across groups. The results of this study will, therefore, offer new perspectives on fall prevention programs with effects on both fear of falling and risks of falls, with increased motivation and adherence from the older adult. In addition, links between actimetry and clinical data for this specific population will be explored. The study by Pereira et al (2014) [10] demonstrated that being active (at least 1125 metabolic expenditure minutes per week) was correlated with a decreased risk of falls [10]. The measurement of actimetry has been shown to be an accurate representation of older adults’ motor activity [55]. However, the use of actimetry to measure physical activity has limitations; certain activities may not be measured properly depending on where the device is worn on the body (eg, wrist, waist) [55]. Moreover, the removal of the device can sometimes be interpreted as a period of inactivity [55]. This latter limitation will be mitigated through the collaboration of the hospital’s care staff, who will notify the research team in the event of any removal of the device. Measuring the evolution of the older adults’ actimetry during and after their rehabilitation program will help develop further knowledge on rehabilitation programs, which has the potential to provide older adults with more adapted rehabilitation programs and decrease the incidence of falls [5].

### Strengths and Limitations

One of the strengths of the study is that it follows the SPIRIT guidelines [26,27] and the PEDro recommendations [33]. It ensures internal and external validity and provides the necessary information to interpret the results of this study. Another strength is the comparison to the standard in rehabilitation for fall prevention in the context of French geriatric hospitals. Regarding outcome measurement, standardized measures have been chosen based on their sensitivity to change [40] to highlight the effect of the intervention. Moreover, results regarding the intervention effectiveness are assessed with standardized scales and with accelerometers, ensuring data triangulation.

This protocol also presents some limitations. Due to the nature of the intervention, double blinding is not possible. However, measures of the outcomes will be blinded and performed by trained assessors to minimize bias. Moreover, stratification on age, gender, fear of falling, and risk of falls will not be possible for the randomization, as the participants will not all be identified at the beginning of the study. However, possible differences between groups at baseline will be tested to consider them in data interpretation. Desirability bias can occur with participants trying to please the assessor. This bias will be the same in both groups. To minimize this bias, primary and secondary outcomes will be assessed by an independent assessor (different from the psychomotor therapist administrating the intervention). This is an exploratory study with significant limitations in terms of sample size, power, and hence, depth of the statistical analysis that can be conducted. However, in the context of a future larger trial (if warranted), an appropriate sample size and power calculation will be undertaken, which would also allow for a more detailed and robust statistical analysis of the data with enhanced validity and integrity of the results.

## Appendix

Multimedia Appendix 1 Ethics approval and funding proof.

## Abbreviations

FES-I: Fall Efficacy Scale–International
MMSE: Mini Mental State Examination
SPIRIT: Standard Protocol Items: Recommendations for Interventional Trials
SPPB: Score Physical Performance Battery
TUAG: Timed Up and Go
