# Supplementary material for: Effects of a Rehabilitation Program Using a Patient-Personalized Exergame on Fear of Falling and Risk of Falls in Vulnerable Older Adults: Protocol for a Randomized Controlled Group Study
Source: JMIR Res Protoc. 2021 Aug 26;10(8):e24665. doi: 10.2196/24665 (PMC8430847; doi:10.2196/24665)
Supplement: Multimedia Appendix 1 [file resprot_v10i8e24665_app1.pdf]

NORTH-WEST COMMITTEE FOR THE PROTECTION OF PERSONS NORTH-WEST I

UNIVERSITY OF ROUEN - MEDICAL/PHARMACEUTICAL FACULTY
Building D - Administration - University Library
22, Boulevard Gambetta - 3rd Floor
76183 ROUEN cedex
+33232888446
@ : cpp.nordouest1@chu-rouen.fr

President: F. BAUER
Vice-President: B. GEFFROY
Secretary General: M. LAURENT

File Number: 19.07.16.46745
National Number: 2019-A00395-52
Internal Ref: MEDIMOOV_01

Mr Professor BELMIN
ASSOCIATION RIVAGES
7, avenue de la République
94200 Ivry-sur-Seine, France

Sir,

The North-Western I Committee for the Protection of Persons (CPP), approved by ministerial order dated 15/05/2018, constituted according to the order of the Director General of the Regional Health Agency of the Haute-Normandie region dated 01/06/2018, has examined the research protocol mentioned in 2° of article L.1121-1 of the CSP (research with minimal risks and constraints) of which you are the coordinating investigator and the promoter, the Association RIVAGES, entitled:

Effects of an adapted physical activity with an interactive game platform on the apprehension of falling and the risk of falling: a randomized controlled study.

This protocol was considered at a meeting on Thursday, 21 November 2019.

The members who deliberated at this meeting were:

College I:
Mrs BAUER(T), Mrs CHAUVET(S), Mrs LEBLANC(T), Mrs CABOURG(S), qualified in the field of biomedical research
Mrs CASTANET(S), paediatrician
Mr LADNER(T), qualified in biostatistics or epidemiology
Ms BRASSEUR(T), general practitioner
Ms BAUER(S), nurse

College II:
Ms. BONNET(T), psychologist
Mmes PANZERI(T), POULET(T) lawyers
Mrs DUPONT(T), Mr SCHAPMAN(T), representative of approved associations of patients or users of the health system.

- The Committee, at its meeting on Thursday, 21/11/2019, requested minor changes to the CNRIPH IS on 25/11/2019.

- The Select Committee which met on 05/12/2019 is not entirely satisfied with the changes made, it requests that the information note concerning the paragraph "Study fees" be amended. The changes were made on the CNRIPH IS on 06/12/2019.
- The select committee of 09/12/2019 considering these modifications and considering that this protocol is in conformity with the 2° of the article L.1121-1 of the Public Health Code, notifies the following final opinion:

FAVOURABLE ASSESSMENT

This opinion delivered on Monday 9th, December 2019 is valid for 2 years, if the research has not begun during this period, it becomes null and void (Article R. 1123-26). The first inclusion must be declared to the CPP as soon as possible.

Documents examined and approved on 21/11/2019:

- Letter requesting an opinion signed and dated 15/07/2019
- Request for advice form signed and dated 12/11/2018
- Additional document requesting an opinion signed and dated 22/07/2019
- EC Declaration of Conformity for Class I medical device dated 08/07/2015
- User manual version 1.3
- Summary version 02 of 25/09/2019
- Certificate of Insurance dated 03/09/2019 with a projected start and end date of
- 01/10/2019 to 01/09/2020
- Test scales MEDIMOOV version 02 of 25/09/2019
- List of investigators version 01 of 11/07/2019 + CVs of Drs BELMIN and LAPIERRE

Documents considered and approved on 05/12/2019:

- Letter of response to the PPC's remarks signed and dated 25/11/2019 Protocol version No. 2 of 22/11/2019
- Summary version no. 2 of 22/11/2019

Documents considered and approved on 09/12/2019:

- Patient Information Letter version 4 dated 06/12/2019

Please believe, sir, in the assurance of my best feelings.

Done in ROUEN, on 13/12/2019.

Professor Fabrice BAUER
President of the CPP NO I

Gérond'if
LE GERONTOPOLE D'ILE-DE-FRANCE

33, rue du Fer à Moulin
75005 Paris

Association Loi 1901

SIRET : 822 452 181 000 16

NAF : 9499Z

Isabelle DUFOUR
Déléguée Générale
isabelle.dufour@gerondif.org

☎ : 01.85.78.10.11

Secrétariat :
Elise ELMACIN
elise.elmacin@gerondif.org

☎ : 01.85.78.10.10
Fax: 01.85.73.56.60
https://www.gerondif.org/

July 2, 2019

Nolwenn Lapierre

<u>Subject</u>: Results of the DIM Longevity and Ageing call for applications

Madam,

I am pleased to inform you that the jury in charge of examining the PhD applications financed within the framework of the DIM, Domain of Major Interest Longevity and Ageing, led by Gérond'if, which I chair, has issued a favorable opinion on the financing of your post-doctoral allowance for a period of 12 months.

I would be grateful if you could send us, by return of e-mail, the contact details of the University manager with whom we will enter into a financial agreement in order to record this financial support.

Congratulations and please accept, Madam, the assurances of my highest consideration.

Yours sincerely,

Isabelle DUFOUR
Chief Delegate
